# Genetic diversity and population structure of the Taigan dog breed

**DOI:** 10.1002/2211-5463.70065

**Published:** 2025-06-17

**Authors:** Kira Bespalova, Anastassiya Perfilyeva, Мamura Begmanova, Assel Zhaxylykova, Arailym Yerzhan, Kanagat Yergali, Almazbek Akunov, Marat Munarbek uulu, Anna Khamchukova, Almira Amirgaliyeva, Yelena Kuzovleva

**Affiliations:** ^1^ Laboratory of Molecular Genetics Institute of Genetics and Physiology Almaty Kazakhstan; ^2^ Salbuurun Federation Bokonbaevo Kyrgyzstan; ^3^ Salymbekov University Bishkek Kyrgyzstan; ^4^ Laboratory of Biocenology and Hunting Management Institute of Zoology Almaty Kazakhstan

**Keywords:** admixture analysis, genetic diversity, population structure, short tandem repeat, single nucleotide polymorphism, Taigan dog

## Abstract

The Taigan is an ancient sighthound breed native to the Tien Shan Mountains in Kyrgyzstan and adapted to hunting at high altitudes and in rough terrain. Previous studies have provided insights into its phylogenetic relationships, but more data are needed to determine whether the Taigan is genetically distinct from related sighthounds. In the present study, we conducted a comprehensive genetic analysis using short tandem repeat markers and high‐density single nucleotide polymorphism array data to assess genetic diversity, population structure and differentiation from other sighthound breeds. The analysis showed high polymorphism and an excess of heterozygosity (*F* = −0.013), indicating a balanced genetic structure. Bayesian clustering identified five genetic clusters among the Taigans, with no dominant lineage, suggesting a diverse gene pool. Principal component analysis and admixture analyses assigned the Taigan to the eastern sighthound group, closely clustered with the Kazakh Tazy. A pairwise *F*_ST_ analysis showed only 17 highly divergent single nucleotide polymorphisms. This number is significantly lower than in recognized and well‐differentiated breed pairs and suggests that genetic differentiation between Kazakh Tazy and Taigan breeds is minimal under current sampling conditions. Further studies with larger data sets are needed to determine the genetic divergence between the Taigan and the Kazakh Tazy.

## Graphical abstract

The Taigan is a rare sighthound from the mountains of Kyrgyzstan. We used DNA markers to study its genetic diversity and compare it with other sighthound breeds. Our findings show that the Taigan shares close genetic ties with the Kazakh Tazy, suggesting a common ancestry shaped by nomadic traditions and regional adaptation.

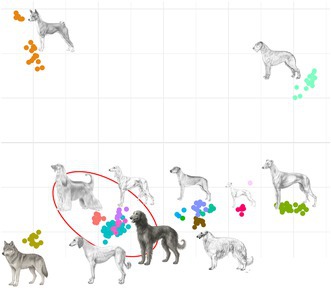

**Abbreviations**
CV: cross‐validation
*F*
: inbreeding coefficient
*H*_e_
: expected heterozygosity
*H*_o_
: observed heterozygosity
HWE: Hardy–Weinberg equilibrium
*N*_a_
: average alleles per locus
*N*_e_
: average effective alleles per locus
PCA: principal component analysis
PIC: polymorphism information content
SNP: single nucleotide polymorphism
STR: short tandem repeat
WGS: whole genome sequencing

Sighthounds are a group of dog breeds selected for their exceptional speed, agility and hunting ability. A recent whole genome sequencing (WGS) study of 123 sighthounds hypothesized that sighthounds have multiple origins, with independent breeding occurring in Africa, Europe and the Middle East [1]. Complex admixture patterns have shaped their genetic diversity. At the same time, although sighthounds from different regions have different evolutionary origins, common selection pressures have simultaneously led to similar phenotypic adaptations. Positive selection signals in these breeds are particularly strong in the areas of bone mineral density, muscle regeneration and cardiovascular efficiency, comprising important physiological traits essential for their endurance and sprinting ability [1]. Although these traits are crucial, different environmental influences have shaped the adaptations of individual breeds.

Among sighthounds, the Taigan, a sighthound native to Kyrgyzstan, has developed unique adaptations to the mountainous landscapes of the Tien Shan region. It is well adapted to hunting in mountainous, rugged terrain at altitudes of 2000–4000 m above sea level [2]. References to the Taigan in ancient Kyrgyz epics, along with skeletal remains from burial mounds that closely resemble the breed, provide strong evidence of its ancient origins [3]. The modern history of the Taigan is closely linked to the social and political changes in Kyrgyzstan and the broader region. In 1964, the Soviet Union established the first breed standard for pedigree dogs and recognized the Taigan as a hunting sighthound. Hunting with sighthounds remained legal in the USSR, although hunters organized in collectives had to surrender a quota of pelts (Fig. 1). After Kyrgyzstan gained independence in 1991, the role of pedigree dog breeding changed. The agricultural reforms of the 1990s, which dissolved the system of collective farming, led to many rural inhabitants returning to a semi‐nomadic lifestyle. For some rural communities, hunting with Taigans once again became the main source of livelihood. In recent years, the urban elite increasingly viewed Taigans as a symbol of national heritage and bred them for prestige. In 1995, the Republican Cynological Council of Kyrgyzstan officially adopted the breed standard for the Taigan [4]. Although the Taigan is recognized by the German Kennel Club, it has not yet been included in the register of the Fédération Cynologique Internationale [5].

**Fig. 1:**
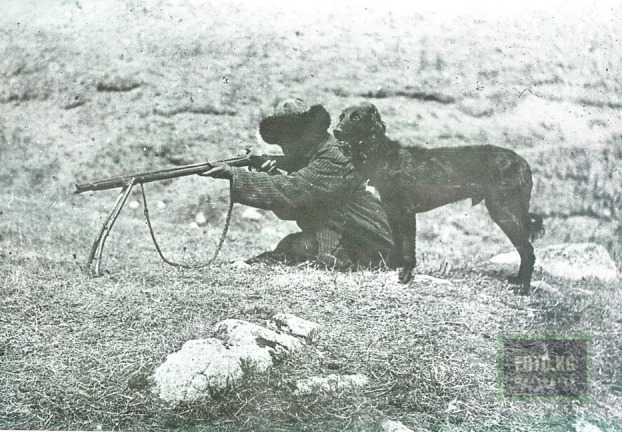
Kyrgyz hunter with Taigan and gun. Photograph: 1910. https://foto.kg/gallery/1891‐ohotnik‐kyrgyz‐s‐Taiganom.html.

One of the key requirements for international recognition is a comprehensive genetic evaluation to determine the distinctiveness of the breed and to clarify its relationship with other sighthounds. Based on the WGS of 15 Taigan dogs, their close phylogenetic relationship with the eastern sighthounds, the Kazakh Tazy and the Afghan Hound, both recognized by the Fédération Cynologique International, has been established [1]. However, despite their common ancestry, these breeds have different adaptations as a result of their respective environments and historical functions. The Kazakh Tazy was primarily used for hunting in open steppe landscapes, whereas the Taigan evolved to withstand the harsh, high‐altitude conditions of mountainous regions. Compared to the Afghan Hound, the Taigan has a denser coat, which is probably an adaptation to the colder temperatures and harsher conditions of the Tien Shan Mountains, whereas the lighter coat of the Afghan Hound is adapted to the relatively warmer and drier climate of Afghanistan [3] (Fig. 2). These morphological and ecological differences suggest adaptive divergence, but the degree of genetic differentiation between the Taigan and its closely related breeds remains unclear. Furthermore, it would be valuable to assess the population structure of Taigan dogs using high‐density single nucleotide polymorphism (SNP) arrays and short tandem repeat (STR) markers, both of which provide complementary insights into genetic diversity and breed differentiation. While SNP arrays enable high‐resolution genome‐wide analysis, STR markers remain a valuable tool for population genetic studies, especially for assessing genetic variability, degree of inbreeding and breed structure. Both STR and SNP array analyses have been widely used for the genetic characterization of various dog breeds and have provided important insights into breed formation, historical gene flow and selection signatures [6, 7, 8, 9, 10, 11, 12, 13, 14, 15, 16, 17, 18, 19, 20, 21]. By integrating both approaches, a more comprehensive understanding of the genetic landscape of Taigan can be achieved, complementing the knowledge gained through WGS.

**Fig. 2:**
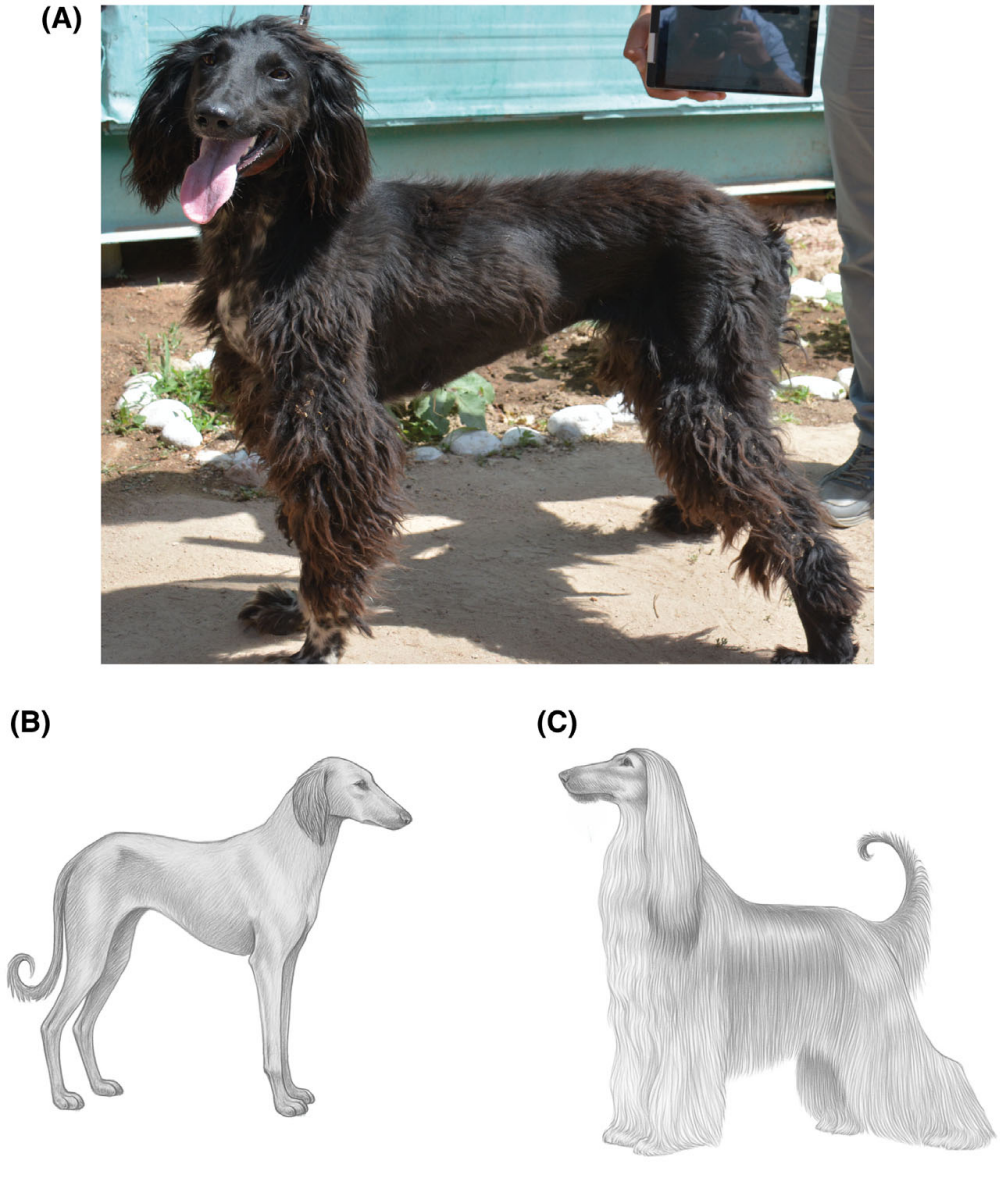
Picture of modern Taigan (A), Kazakh Tazy (B) [37] and Afghan Hound (C) [38]. Illustrations redrawn by the authors based on breed standards from Refs [37, 38].

To address these gaps, we conducted a comprehensive genetic analysis of the Taigan using STR and SNP markers to clarify its genetic status and assess its population structure. Additionally, we quantified genetic differentiation between the Taigan and related sighthounds, identifying genomic regions shaped by selection, genetic drift or geographic isolation. These findings contribute to the broader understanding of sighthound diversity and breed differentiation, offering critical insights into the Taigan's potential recognition as a distinct breed.

## Materials and methods

### Sample collection and DNA extraction

All applicable international, national and institutional guidelines for the care and use of animals were strictly followed. All procedures were approved by the Bioethics Committee of the Institute of Molecular Biology and Biochemistry named after M. A. Aitkhozhin, Almaty, Kazakhstan (#1, 18 August 2023).

The DNA samples were obtained by taking buccal swabs and/or blood samples at a special event in the Kyrgyz Republic. In total, 43 samples were collected from Taigan dogs (Fig. S1). The evaluation of conformity with the breed standard was carried out by the experts. Only dogs that were confirmed to conform to the official breed standard were selected for sampling. All owners gave their informed written consent to use samples from their dogs for genetic studies. DNA was extracted using QiaAmp DNA extraction kits in accordance with the manufacturer's protocol (Qiagen, Valencia, CA, USA).

### STR genotyping

In total, 19 STR markers (AHTk211, CXX279, REN169O18, INU055, REN54P11, INRA21, AHT137, REN169D01, AHTh260, AHTk253, INU005, INU030, amelogenin, FH2848, AHT121, FH2054, REN162C04 AHTh171 and REN247M23) recommended by the International Society for Animal Genetics were amplified using a commercial Canine ISAG STR Parentage Kit (Thermo Fisher Scientific, Waltham, MA, USA) for a total of 43 DNA samples. Genotyping was performed using the SeqStudio™ Genetic Analyzer (Thermo Fisher Scientific). The STR were processed with genemapper, version 6 (Thermo Fisher Scientific) and checked manually.

### SNP genotyping

Of the 43 Taigan samples collected, eight dogs were selected for SNP genotyping based on expert evaluations by experienced cynologists. The selection criteria prioritized dogs with the most distinct breed‐specific morphological characteristics to ensure that the core traits of the Taigan breed are represented in the genomic analysis. Samples were genotyped using a Canine HD Genotyping BeadChip (Illumina, San Diego, CA, USA). Genotyping was performed on the iScan system (Illumina) using genomestudio software (Illumina) and data were exported in plink, version 1.9, format [22].

### SNP data processing and quality control

The SNP genotype data of Taigan were merged with publicly available SNP array data of 14 wolves and 173 dogs from 15 sighthound breeds (Table 1). For each breed, we preferentially selected individuals from native, country‐of‐origin populations whenever such metadata were available. The sample code and corresponding breed are listed in Table S1.

**Table 1:** List of sighthound breeds with sample sizes and geographic origins.

Breed	Number of sample	Group
Afghan Hound	11	East
Basenji	20	African
Borzoi	12	North
Greyhound	28	West
Ibizan Hound	1	Mediterranean
Irish Wolfhound	18	North
Italian Greyhound	6	West
Kazakh Tazy	39	East
Pharaoh Hound	2	Mediterranean
Portuguese Podengo	1	Mediterranean
Rhodesian Ridgeback	10	African
Saluki	7	East
Scottish Deerhound	1	North
Whippet	6	West

The SNP datasets were merged using the ‐‐merge‐list command to create a combined dataset of 156 566 SNPs. The quality filtering was applied to the merged dataset: SNPs with a call rate < 95%, a minor allele frequency < 0.05, and those deviating from Hardy–Weinberg equilibrium (HWE) (*P* < 0.01) were filtered out. In total, 79 158 SNPs were removed because of missing genotype data, 49 765 SNPs did not pass the HWE test and 3082 SNPs had a minor allele frequency below the threshold. After applying these filters, 20 486 SNPs and 184 samples remained. To ensure marker independence for population structure analyses, linkage disequilibrium (LD) pruning was performed using PLINK and the ‐‐indep‐pairwise 50 5 0.2 command. This procedure excluded SNPs with strong LD and resulted in a final dataset of 15 315 adjusted SNPs, which was used for further analyses. The reduction in SNP number was primarily due to platform differences between datasets and the pruning of high LD markers for principal component analysis (PCA) and ADMIXTURE analyses. The remaining markers were well distributed across the genome and suitable for population‐level inference.

### Genetic diversity and structure analysis based on the STR data

The allele frequencies of 18 STR loci were used to determine the average alleles per locus (*N*_a_), average effective alleles per locus (*N*_e_), observed heterozygosity (*H*_o_), expected heterozygosity (*H*_e_) and inbreeding coefficient (*F*), polymorphism information content (PIC) and deviations from HWE using genaiex 6.5 [23, 24]. Bonferroni corrections were applied for HWE estimation. Bayesian clustering was performed using structure, version 2.3.4, to identify internal structure in a population (clustering) [25]. Simulations were performed under an admixture model with correlated allele frequencies using a burn‐in period of 5000 iterations followed by 10 000 MCMC replicates. The optimal number of clusters (best *K*) was determined using the Δ*K* method as previously described by Evanno *et al*. [26].

### PCA and admixture analyses based on the SNP data

A filtered SNP dataset was used for the ADMIXTURE analysis with the admixture software [27]. A range of *K* from 2 to 10 was examined, with 10 cross‐validation replicates performed for each *K* value. Cross‐validation (CV) was performed for each *K* value (‐‐cv flag) and the optimal *K* was determined based on the minimum CV error. A PCA of the filtered data was performed using plink, version 1.9, and the ‐‐pca command [7, 12]. The resulting data of both analyses were visualized in r with the package ggplot2 [28].

### *F*_ST_‐based genomic divergence analysis based on the SNP data

To evaluate genomic differentiation between the Kazakh Tazy and Taigan breeds, pairwise *F*_ST_ values were calculated for autosomal and X chromosomes using vcftools [29]. *F*_ST_ statistics were calculated for each SNP and Bonferroni correction was applied to account for multiple testing. The genome‐wide distribution of differentiation was visualized using a Manhattan plot. Data processing and visualization was performed in r using the ggplot2 package [28]. SNPs with values of *F*_ST_ > 0.5 and statistically significant *P*‐values were considered to indicate substantial genetic differentiation. We used a conservative *F*_ST_ threshold of 0.5 to highlight the most differentiated loci. Although moderate levels of differentiation (e.g. *F*_ST_ > 0.25) may be a result of genetic drift or limited gene flow, values above 0.5 typically indicate near fixation of alternative alleles and are often associated with divergent selection or long‐term reproductive isolation. This threshold has often been used in genome scan studies to identify outlier loci subject to selection [30, 31]. To quantify genetic differentiation between selected dog breeds, we conducted pairwise *F*_ST_ analysis. For each breed pair, we extracted shared outlier SNPs and constructed an intersection matrix to assess overlap across comparisons.

## Results

### Diversity analysis based on the STR data

In total, 135 alleles were detected at the 18 STR loci (Table 2). The highest number of alleles was observed for AHT121 (*N* = 11 alleles per locus), whereas the lowest number was observed for AHTk211 (*N* = 4 alleles per locus). The average *N*_a_ across all loci was 7.5, whereas the mean *N*_e_ was 3.95, ranging from 1.48 for REN247M23 to 5.68 for FH2054. *H*_o_ ranged from 0.35 for REN247M23 to 0.88 for REN169D01, with an average value of 0.72 for all loci. *H*_e_ ranged from 0.32 for REN247M23 to 0.82 for CXX0279 and AHT121, with an average value of 0.71. *F* varied from −0.16 for REN162C04 to 0.11 for INU005, with an overall mean of −0.013, indicating a slight excess of heterozygotes in the population. The mean value of PIC was estimated to be approximately 68%, ranging from 31% for REN247M23 to 81% for FH2054 (Table 3). PIC above 60% was observed for all STR markers except four loci (REN247M23, INU005, AHTk253 and AHTk211), whereas a value above 80% was observed only for FH2054.

**Table 2:** Polymorphism of 18 STR markers of the Taigan. *I*, Shannon's information index.

Locus	*N*	*N*_a_	*N*_e_	*I*	*H*_o_	*H*_e_	*F*	PIC
AHTk211	43	4	2.127	1.006	0.605	0.530	−0.141	0.49
CXX0279	43	7	5.620	1.826	0.791	0.822	0.038	0.80
REN169O18	43	8	4.778	1.737	0.767	0.791	0.029	0.76
INU055	43	7	3.773	1.478	0.698	0.735	0.051	0.69
REN54P11	43	6	3.732	1.470	0.674	0.732	0.079	0.69
INRA21	43	9	3.625	1.614	0.698	0.724	0.037	0.69
AHT137	43	10	4.905	1.844	0.837	0.796	−0.052	0.77
REN169D01	43	10	5.430	1.861	0.884	0.816	−0.083	0.79
AHTh260	43	8	3.287	1.549	0.744	0.696	−0.070	0.67
AHTk253	43	7	2.787	1.257	0.628	0.641	0.021	0.57
INU005	42	7	2.600	1.221	0.548	0.615	0.110	0.59
INU030	43	6	3.529	1.419	0.674	0.717	0.059	0.67
FH2848	43	7	4.729	1.708	0.791	0.789	−0.003	0.76
AHT121	43	11	5.430	1.969	0.791	0.816	0.031	0.80
FH2054	42	8	5.681	1.869	0.857	0.824	−0.040	0.81
REN162C04	43	6	2.982	1.383	0.767	0.665	−0.155	0.63
AHTh171	43	9	4.549	1.783	0.837	0.780	−0.073	0.76
REN247M23	43	5	1.479	0.678	0.349	0.324	−0.078	0.31
Mean		7.5	3.947	1.537	0.719	0.712	−0.013	0.68
SE	0.076	0.43	0.297	0.080	0.031	0.030	0.018	0.03

**Table 3:** Summary of chi‐squared tests for HWE. ns, not significant.

Pop	Locus	Chi‐square	*P*	Significance
Pop1	AHTk211	3.538	0.739	ns
Pop1	CXX0279	22.248	0.385	ns
Pop1	REN169O18	17.585	0.936	ns
Pop1	INU055	18.999	0.585	ns
Pop1	REN54P11	17.116	0.312	ns
Pop1	INRA21	31.976	0.660	ns
Pop1	AHT137	43.496	0.536	ns
Pop1	REN169D01	30.246	0.955	ns
Pop1	AHTh260	22.308	0.767	ns
Pop1	AHTk253	28.158	0.136	ns
Pop1	INU005	22.026	0.398	ns
Pop1	INU030	9.419	0.855	ns
Pop1	FH2848	22.548	0.369	ns
Pop1	AHT121	55.761	0.446	ns
Pop1	FH2054	37.082	0.117	ns
Pop1	REN162C04	9.685	0.839	ns
Pop1	AHTh171	37.748	0.389	ns
Pop1	REN247M23	11.395	0.328	ns

The Hardy–Weinberg test performed separately for each locus showed no deviation from the expected frequencies after Bonferroni correction (*P* > 0.05).

### structure analysis based on the STR dataset

**Fig. 3:**
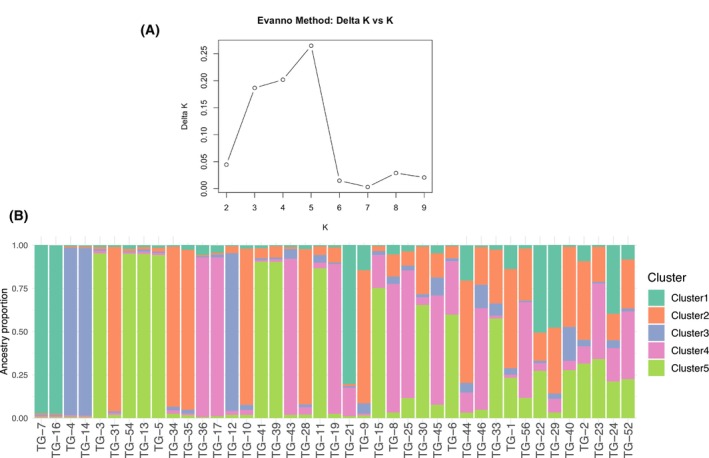
Genetic structure of the Taigan dogs. Bayesian clustering on the STR dataset of 41 dogs performed with structure, version 2.3.4, after correction by Evanno *et al*. (A) Delta *K* plot showing optimal number of clusters. (B) bar plots where each dog is represented by a single vertical line. Dogs are sorted along the *X* axis according to their proportion in the dominant cluster.

The Bayesian clustering analysis was performed to assess the genetic structure of 41 Taigan dogs across 18 STR loci. The analysis indicated that *K* = 5 provided the best clustering resolution (Fig. 3). Five clusters reflect intrabreed structure.

The different clusters had relatively balanced proportions, with no single genetic component dominating the population as a whole. Most individuals exhibited admixture patterns, with varying contributions from multiple clusters rather than exclusive membership to one. The most represented component was cluster 5 (28.3%), while cluster 3 was the least represented (10.0%) (Table 4).

**Table 4:** Allele frequency divergence.

Cluster	Proportion of membership	Average distance	*F*_ST_
1	0.130	0.5452	0.3360
2	0.259	0.7665	0.0069
3	0.100	0.6488	0.2342
4	0.227	0.5838	0.2341
5	0.283	0.6981	0.1211

### Genetic diversity and population structures based on the SNP dataset

**Fig. 4:**
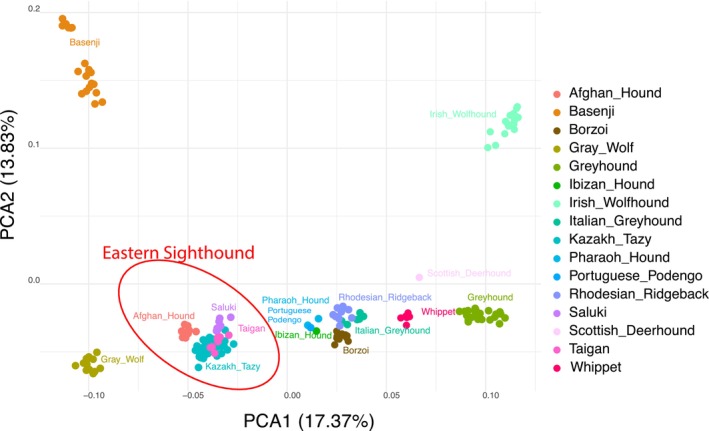
Genetic structure of sighthound breeds based on PCA. The group of eastern sighthounds are indicated with a red oval.

PCA was conducted on eight Taigan dogs along with 173 dogs representing 15 sighthound breeds and two wolves to assess their genetic relationships and population structure within the broad sighthound group (Fig. 4). The PCA showed that Taigans were closely clustered with sighthounds of the East group, including Saluki, Afghan Hound and Kazakh Tazy.

**Fig. 5:**
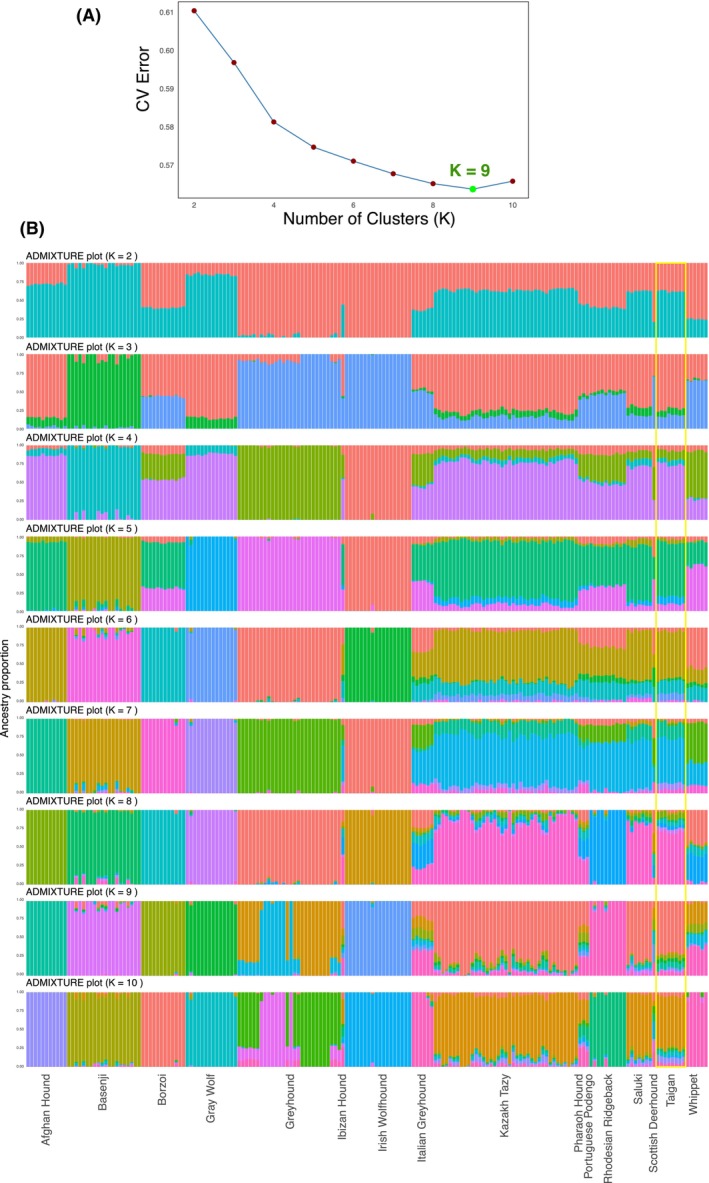
admixture analysis in sighthounds dogs. (A) Cross‐validation error by *K*. (B) Bar plots where each vertical bar represents an individual dog. Taigan dogs are indicated with a yellow rectangle.

To assess admixture patterns between the sighthound breeds, we performed an ADMIXTURE analysis (Fig. 5). At lower *K* values, the Taigans showed a similar genetic mixing pattern to most sighthounds. However, as the *K* value increased (from 5 to 10), the Taigans, along with other sighthounds of the East group, became genetically distinct from sighthounds of other geographic groups. This pattern remained consistent up to *K* = 10, with one notable exception: the Afghan Hound, which diverged from the other eastern sighthounds at *K* = 6. The lowest CV error was observed at *K* = 9 (CV = 0.564) (Fig. 5A).

### Genomic differentiation between Kazakh Tazy and Taigan

**Fig. 6:**
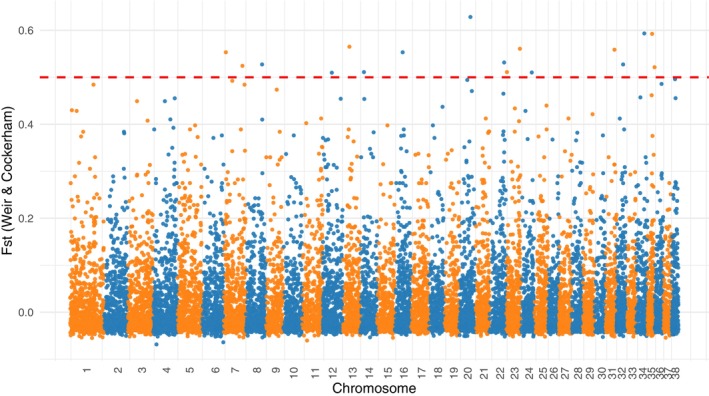
Manhattan plot of *F*_ST_ (Kazakh Tazy and Taigan). Chromosomes were distinguished by alternating colors, and a reference line at *F*_ST_ = 0.5 indicated regions with high genetic differentiation.

Although the ADMIXTURE analysis showed similar genetic patterns for Taigan, Kazakh Tazy and Saluki, the PCA was more sensitive and distinguished the Saluki from this group based on their genetic variation, while Kazakh Tazy and Taigan remained tightly clustered. To determine whether there is genetic differentiation between Kazakh Tazy and Taigan and to identify the specific genes involved, we performed an *F*_ST_ analysis. Based on 15 315 SNPs, the genome‐wide Weir and Cockerham mean *F*_ST_ was 0.022204, and the weighted *F*_ST_ was 0.027112, indicating an overall low level of genetic differentiation between these two breeds. A total of 17 statistically significant variants were identified (Fig. 6).

To determine whether the extent of genomic divergence between Kazakh Tazy and Taigan is unusually low, we performed the same outlier‐based *F*_ST_ analysis for several other breed pairs using the same set of filtered and LD‐cut SNPs (15 315 loci). The results are summarized in Table 5. This comparative analysis revealed that the number of SNPs with high divergence (*F*_ST_ > 0.5, *P* < 0.05) was significantly higher in recognized breed pairs such as Saluki vs. Afghan Hound (814 SNPs) and Greyhound vs. Whippet (657 SNPs) than in Kazakh Tazy vs. Taigan (17 SNPs). This supports the conclusion that the observed genetic differentiation between Tazy and Taigan is minimal under the current sampling conditions.

**Table 5:** Comparative summary of outlier SNPs across breed pairs.

Breed pair	Total SNP	Outlier SNP	% Outlier
Kazakh Tazy vs. Taigan	15 250	17	0.11
Kazakh Tazy vs. Saluki	15 238	102	0.67
Saluki vs. Afghan Hound	13 878	814	5.87
Greyhound vs. Whippet	13 953	657	4.71

## Discussion

In this study, we conducted a comprehensive genetic analysis of the Taigan to assess its genetic diversity, population structure, and differentiation from closely related sighthound breeds. Using a combination of STR markers and high‐density SNP array data, we aimed to supplement existing findings and provide a more detailed understanding of the breed's genetic status and genetic history.

The Taigan breed demonstrated a high degree of genetic variability, as shown by the observed heterozygosity and the informativeness of the STR markers. This level of diversity is comparable to that of the Kazakh Tazy and higher than in many other domestic dog breeds such as the German Shepherd or the Italian Greyhound [32, 33, 34]. The increased heterozygosity in Taigans may reflect a combination of factors, including historical breeding practices, geographic isolation, regional lineage divergence, or genetic drift. The traditional breeding practices of Taigan owners often focused on functional traits such as endurance, hunting ability and temperament rather than standardized morphology. Breeding decisions were usually made within local communities, sometimes based on oral traditions or personal experience, rather than centralized registries or formal pedigrees [4]. This may have helped to preserve the different genetic lines within the breed. Geographical isolation also played an important role. The Taigan was historically bred in the mountainous regions of Kyrgyzstan, where communities were separated by rugged terrain, limited infrastructure and seasonal inaccessibility. These conditions have reduced gene flow between groups and encouraged the development of different local lineages. In some cases, lineages persisted across generations in single valleys or settlements, further reinforcing differentiation through founder effects or restricted mate choice.

To improve the resolution of the analysis, we performed SNP genotyping on a cohort of eight Taigans and compared it with data from 15 sighthound breeds. As already shown based on the WGS data [1], the PCA analysis placed the Taigans to the East group and clearly distinguished this group from the other groups. Interestingly, in a previous study, the Kazakh Tazy was genetically distinct from the Saluki and Afghan Hound in the PCA analysis [34]. In our study, they also formed a somewhat separate cluster from other eastern sighthounds, with the exception of the Taigans, which remained closely grouped with them. The admixture patterns showed that only the Afghan Hound had a unique genetic profile when *K* was increased, while the Kazakh Tazy, Taigan and Saluki had very similar genetic patterns, suggesting a common ancestry.

Overall, PCA and admixture analyses indicate a close relationship between the Kazakh Tazy and the Taigan, which is not unexpected given the long history of cultural and economic connections between Kazakhstan and Kyrgyzstan. These connections were shaped by centuries of nomadic migrations, transhumance and shared pastoralism. As early as the 2nd century BC, the Great Silk Road facilitated the movement of people, livestock and cultural practices across Central Asia, including the exchange and cross‐breeding of greyhound species used for hunting and protection [35, 36]. Seasonal herding routes through the Tien Shan Mountains and the Kazakh steppe ensured continuous interaction between Kyrgyz and Kazakh herders well into the 20th century. The regular exchange of animals, coupled with overlapping ecological functions and shared selection pressures, likely contributed to the observed genetic similarity between the Kazakh Tazy and the Taigan.

However, given their adaptation to different ecological conditions, it is possible that local selection pressures have contributed to the subtle genetic differences between these breeds. To determine whether the Kazakh Tazy and Taigan represent distinct genetic lineages, we performed a pairwise *F*_ST_ analysis. The number of highly divergent SNPs was significantly higher in recognized and well‐differentiated breed pairs such as the Saluki and the Afghan Hound (814 SNPs) and the Greyhound and the Whippet (657 SNPs) compared to only 17 such SNPs between the Tazy and the Taigan. This finding supports the interpretation that genetic differentiation between Kazakh Tazy and Taigan is minimal under current sampling conditions.

Given the limited sample size and possibly narrow geographic origin of the Taigan individuals, the observed genetic homogeneity should be interpreted with caution. It remains possible that a more comprehensive sample, particularly with field expeditions to remote regions of Kyrgyzstan where native Taigans are traditionally bred and cared for, could reveal patterns of population structure that were not apparent in the present dataset.

In conclusion, the present study is the first to investigate the genetic diversity and structure of the national Kyrgyz sighthound breed, Taigan, based on STR markers and SNP array data. Our results showed a high level of genetic diversity and a complex genetic structure within the breed. In a broader analysis together with sighthounds from different geographical regions, the Taigan showed a genetic variation comparable to that of other eastern sighthounds such as the Kazakh Tazy, Afgan Hound and Saluki. However, the very close clustering between Taigans and Kazakh Tazy raises the question of whether the geographical barriers of the Tien Shan Mountains and adaptation to different hunting conditions have contributed to the genetic divergence of these two breeds. Therefore, further studies with a much larger data set are essential to accurately determine the degree of genetic divergence between Taigans and Kazakh Tazy.

This study is limited by the small sample size. These individuals may not represent the full genetic diversity of the breed, especially that of the native Taigans in the remote regions of Kyrgyzstan. The geographic range of the sample was small, and broader field‐based efforts are needed to capture regional variation.

Another limitation of the present study is the lack of comparable STR genotype data for other dog breeds. Access to such data would allow a more robust contextualization of the observed genetic patterns. This remains a priority for future research.

## Conflicts of interest

The authors declare that they have no conflicts of interest.

## Peer review

The peer review history for this article is available at https://www.webofscience.com/api/gateway/wos/peer‐review/10.1002/2211‐5463.70065.

## Author contributions

AP and KB contributed to writing the original draft, reviewing and editing, supervision and bioinformatic analysis. KB contributed to visualization. AAk, MM and AK contributed to sample collection. MB and AAm contributed to formal analysis. AP, KB AZ, AY, YK and KY contributed to methodology. All authors have read and approved the final version of the manuscript submitted for publication.

## Supporting information


**Fig. S1.** Taigan dogs examined in this study.

**Table S1.** FID and IID.

## Data Availability

The SNP data of eight Taigan dogs are deposited in the Dryad open data publishing platform and can be accessed at http://datadryad.org/share/EJo7UsMHI08o8r0JJDgKu1ylsKcr4TI4bzw2KfvM7YI.
