# Supplementary figures and images for: Genetic diversity and population structure of the Taigan dog breed
Source: FEBS Open Bio. 2025 Jun 17;15(9):1520–31. doi: 10.1002/2211-5463.70065 (PMC12401176; doi:10.1002/2211-5463.70065)

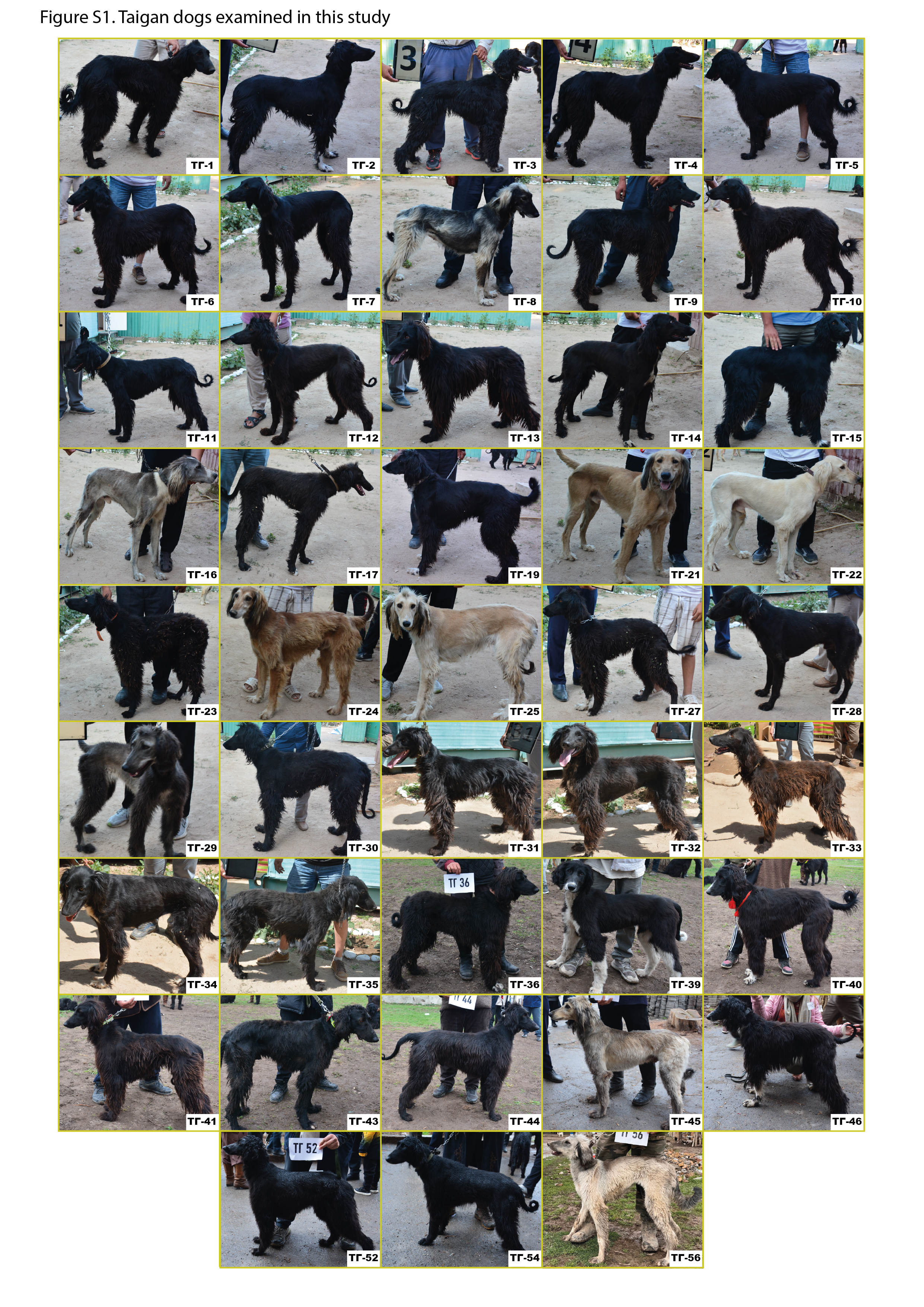

Supplement: Supplementary file 1 — Fig. S1. Taigan dogs examined in this study. [file FEB4-15-1520-s002.png]
